# New Objective Refraction Metric Based on Sphere Fitting to the Wavefront

**DOI:** 10.1155/2017/1909348

**Published:** 2017-09-20

**Authors:** Mateusz Jaskulski, Andreí Martínez-Finkelshtein, Norberto López-Gil

**Affiliations:** ^1^Facultad de Óptica y Optometría, University of Murcia, Murcia, Spain; ^2^Department of Mathematics, University of Almería, Almería, Spain

## Abstract

**Purpose:**

To develop an objective refraction formula based on the ocular wavefront error (WFE) expressed in terms of Zernike coefficients and pupil radius, which would be an accurate predictor of subjective spherical equivalent (SE) for different pupil sizes.

**Methods:**

A sphere is fitted to the ocular wavefront at the center and at a variable distance, *t*. The optimal fitting distance, *t*_opt_, is obtained empirically from a dataset of 308 eyes as a function of objective refraction pupil radius, *r*_0_, and used to define the formula of a new wavefront refraction metric (MTR). The metric is tested in another, independent dataset of 200 eyes.

**Results:**

For pupil radii *r*_0_ ≤ 2 mm, the new metric predicts the equivalent sphere with similar accuracy (<0.1D), however, for *r*_0_ > 2 mm, the mean error of traditional metrics can increase beyond 0.25D, and the MTR remains accurate. The proposed metric allows clinicians to obtain an accurate clinical spherical equivalent value without rescaling/refitting of the wavefront coefficients. It has the potential to be developed into a metric which will be able to predict full spherocylindrical refraction for the desired illumination conditions and corresponding pupil size.

## 1. Introduction

Objective wavefront refraction is a computational technique which can be used to obtain a spherocylindrical prescription that best corrects the subject's vision from a single measurement of ocular wavefront aberrations [1, 2]. The goal is to match the clinical subjective refraction, which has long been the gold standard in optometric practice [1–6], in spite of being a lengthy procedure of relatively poor precision (95% limits of interexaminer agreement of the spherical equivalent 0.62 to 0.75D [4–7]—roughly twice the value reported for wavefront refractions) [4, 7, 8].

In a perfect optical system, a spherical wavefront from an object point should converge to a single point at the desired image location, such as the retina of the eye. In the presence of optical aberrations, however, the wavefront becomes distorted from its spherical shape which degrades the quality of the retinal images [7]. The wavefront error (WFE) is the optical path difference between the aberrated and the ideal, unaberrated wavefront. Because the WFE is measured for the whole area of the pupil, wavefront aberrometers are especially useful for the evaluation of refractive surgery cases, customized ablations, orthokeratology, and similar applications [4, 9, 10]. In daily optometric practice, however, wavefront refraction with these devices tends only to be used to estimate a starting point for subjective refraction [11].

Objective wavefront refraction is based on fitting a reference wavefront produced by an optimum, spherocylindrical lens to a two-dimensional ocular wavefront aberration function measured for a subject's eye. As described by Thibos et al. [7], there are two main categories of methods (hereafter called “metrics”) of finding such spherocylindrical lens.

The first category, which they refer to as “refraction based on the principle of equivalent quadratic” is based on fast and relatively simple computations of Zernike coefficients, which are used to describe the wavefront aberration function in the plane of the pupil. The second category, called “refraction based on maximizing optical or visual quality” is based on far more computationally intensive calculations of optical transfer functions and analyses of images in the plane of the retina using Fourier or geometrical optics. For instance, the VSOTF (visual Strehl ratio calculated from the optical transfer function [7, 12–15]) method can easily take a modern machine 1000 times longer to compute (e.g., 500 ms), as it involves many intermediate steps, such as calculations of normal and diffraction-limited pupil functions, their Fourier transforms, and ratio.

Additionally, the image-plane metrics are no longer directly tied by means of a mathematical formula to basic ocular aberrations, such as defocus, astigmatism, or SA, which are fundamental at the time of prescribing a refraction correction. All these methods from both categories have been widely used to predict subjective refraction not only in normal eyes studies [12–15] but also in peripheral refraction studies [16, 17], in eyes that have undergone refractive surgery [18], contact lenses [19], and even to study the accommodation response [20–22], among many others.

Although the continued development of aberrometers has made wavefront refraction a fast procedure with even better repeatability and precision [1, 4, 7], the accuracy of the technique is still a problem without a simple solution. Thibos (Indiana University) illustrated this by saying “We are aiming a gun (objective refraction) that does not shoot straight at a moving target (subjective refraction).” The “gun” does not shoot straight because objective refraction depends on the metric that is used to obtain it from the WFE, and these are known to suffer from bias [7]. On the other hand, the “target” is moving because subjective refraction changes with pupil size, especially in the presence of spherical aberration (SA), and depends on the level of illumination during measurement [23, 24]. Consequently, the spherocylindrical correction obtained via subjective refraction in clinical conditions does not exactly correspond to the best correction obtained using an aberrometer under different conditions (target size, illumination, cycloplegia, and so forth).

The purposes of the present study were to develop an objective refraction metric based on Zernike coefficients and pupil radius, which would be an accurate predictor of clinical, subjective refraction, and to address the variability between subjective and objective wavefront refraction. We propose a relatively simple pupil-plane formula of a metric that can provide a clinician with an accurate refraction value for a known pupil radius that the subject will typically have. Image-plane metrics were not considered in the present study, which does not in any way deny their huge usefulness in fundamental research, image processing, and other applications. In addition, given that the equivalent sphere is, in most cases, the most important value in the refraction, the study is limited for the sake of simplicity to the prediction of the SE. At the end of the Introduction section, formulas to extend the methodology and to apply it to the whole spherocylindrical refraction are provided ((14) and (15)).

## 2. Methods

### 2.1. Overview of the Datasets

The database of eyes of the present study is an amalgamation of wavefront and subjective refraction data from 4 independent, previously published datasets; the summary of which is presented in Table 1. All subjects were free of any kind of ocular disease and have never had refractive surgery. The data consisted of pupil diameters, signed Zernike coefficients through the fifth or sixth order, and subjective refraction data (sphere, axis, and cylinder) for each individual eye. Subjective refractions were in all cases performed manually, starting from autorefraction, using the standard optometric protocol of maximum plus, to the best visual acuity.

Out of the whole database of 2560 eyes collected by Salmon and van de Pol [25], only the A dataset was included because it contained subjective refraction data compatible with the present study. The eyes were not dilated, and the subjective refraction pupil radius was not known. In case of the H dataset, the eyes were dilated with 1% tropicamide, and the subjective refractions were performed at the same pupil size as the wavefront measurements. In case of the M dataset, the eyes were not dilated and the subjective refraction pupil radius was not known. Six eyes out of 180 had to be excluded from the original dataset, because an in-depth data analysis revealed that the subjects did not perform the accommodation task of the original study correctly. Together, the three datasets formed the AHM dataset of 308 eyes, which was used to develop the objective refraction metric proposed by the present study.

The IND dataset of 200 eyes was used to independently validate the results because the methodology to obtain the data was distinct. After performing initial subjective refractions, accommodation was paralyzed with 0.5% cyclopentolate. The eyes were then optimally corrected for astigmatism, and their hyperfocal points were conjugated with the retina with trial lenses. This correction was worn by the subjects during the subsequent aberrometry. This experimental design emphasized the effects of higher-order aberrations by minimizing the presence of uncorrected second-order aberrations.

The effects of longitudinal chromatic aberration between the wavelength of the infrared measurement beam and visible light, depth of diffuse reflection of infrared light in the choroid [27], and any other internal offsets of the apparatus were taken into account in the data.

### 2.2. Traditional Pupil-Plane Metrics of Objective Wavefront Refraction

The two metrics most widely used in practice, which belong to the category of *wavefront refraction based on the principle of equivalent quadratic*, are paraxial curvature matching at the pupil center (paraxial or Seidel refraction) and paraboloid least squares fitting over the full pupil area (minimum RMS or Zernike refraction) [7]. In both cases, the equivalent sphere is computed from Zernike coefficients *C*_*n*_^*m*^ and pupil radius *r*_0_. Aberrometers typically express the wavefront as an expansion of coefficients up to the sixth order [28].

Zernike refraction (hereafter called the “minRMS metric,” with equivalent sphere *M*_minRMS_) takes into account the Zernike higher-order aberrations (HOA). For this metric, the equivalent sphere is computed as follows:

(1) $$\begin{array}{c} M_{minRMS}=\frac{-4\sqrt{3}C_{2}^{0}}{r_{0}^{2}}, \end{array}$$

where *C*_2_^0^ is the Zernike defocus coefficient and *r*_0_ is the actual pupil radius.

The minRMS metric fits a paraboloid of revolution to the measured ocular wavefront in such a way that the root mean square error between the two is minimized for the whole area of the pupil. It was found to exhibit a systematic, myopic bias of roughly 0.4D [7] and becomes more myopic in the presence of large values of SA [29, 30].

Paraxial refraction (hereafter called the “paraxial metric,” with equivalent sphere *M*_paraxial_) takes into account only the curvature of the wavefront at the pupil center. It is not affected by SA because it does not take into account the peripheral rays. There is evidence, however, that for large pupils, high-contrast objects (as is the case in night vision), and in presence of fourth-order SA, the refractive error may become negative as the eye becomes more myopic [31]. In this case, the paraxial metric yields a hyperopic prediction of subjective refraction.

(2) $$\begin{array}{c} M_{paraxial}=\frac{-4\sqrt{3}C_{2}^{0}+12\sqrt{5}C_{4}^{0}-24\sqrt{7}C_{6}^{0}}{r_{0}^{2}}, \end{array}$$

where *C*_4_^0^ and *C*_6_^0^ are, respectively, the fourth- and sixth-order Zernike spherical aberration coefficients, which contribute to the central curvature of the wavefront because they are *balanced* [32]. This gives rise to a difference of the values of equivalent spheres predicted by both metrics when SA is present in the eye.

In the absence of higher-order aberrations, minRMS and paraxial refractions are identical [13]. Both metrics give predictions of equivalent spheres that match for small pupils, and Campbell [6] reported their excellent agreement with subjective refractions for 4 mm pupils. Both metrics can be expressed by a more general formula in (3). For example, the minRMS equivalent sphere formula from (1) can be obtained from it when *n* = 1.

(3) $$\begin{array}{c} M_{paraxial}=\frac{2}{r_{0}^{2}}\overset{\infty }{\underset{n=1}{\sum }}{\left(-1\right)}^{n}\frac{\left(n+1\right)!}{\left(n-1\right)!}\sqrt{2n+1}C_{2n}^{0}, \end{array}$$

where *C*_2*n*_^0^ is the radially symmetrical Zernike coefficients of the wavefront, *r*_0_ is the actual pupil radius, and *n* is the Zernike order.

In the case of aberrated eyes, Thibos et al. [7] suggest that the clinical subjective refraction should lie somewhere between the paraxial and minRMS refractions, which is in agreement with other reports [14, 15].

### 2.3. Analytical Derivation of the New Objective Wavefront Refraction Metric

To compute an equivalent sphere from a two-dimensional wavefront aberration function, it is first expressed in terms of Zernike polynomials [28].

(4) $$\begin{array}{c} W\left(r\right)=\overset{\infty }{\underset{n=0}{\sum }}C_{2n}^{0}Z_{2n}^{0}\left(\frac{r}{r_{0}}\right), \end{array}$$

where *C*_2*n*_^0^ is the Zernike coefficients of the wavefront, *ρ* = *r*/*r*_0_ is the normalized distance from the origin in the pupil plane, *r*_0_ is the actual pupil radius, and *r* ∈ [−*r*_0_, *r*_0_]. *Z*_2*n*_^*m*^ is a radially symmetric basis functions.

(5) $$\begin{array}{c} Z_{2}^{0}\left(\rho \right)=\sqrt{3}\left(2\rho^{2}-1\right),\\ Z_{4}^{0}\left(\rho \right)=\sqrt{5}\left(6\rho^{4}-6\rho^{2}+1\right),\\ Z_{6}^{0}\left(\rho \right)=\sqrt{7}\left(20\rho^{6}-30\rho^{4}+12\rho^{2}-1\right),\ldots . \end{array}$$

For the sake of simplicity, the present study is focused on finding the equivalent sphere, the derivation is limited to terms with radial symmetry (*m =* 0, and *n* is even), and the coefficients are truncated after the sixth order [32, 33].

The equivalent sphere is found by approximating the wavefront in (4) by a sphere of radius *R*, centered at (*R*, 0, 0).

(6) $$\begin{array}{c} S\left(r\right)=W\left(0\right)+R-\sqrt{R^{2}-r^{2}}=W\left(0\right)+\frac{r^{2}}{R+\sqrt{R^{2}-r^{2}}}. \end{array}$$

The equivalent sphere, *M* = −1/*R*, is expressed as a function of both the Zernike coefficients and the pupil radius *r*_0_. One simple way to approximate the wavefront by a sphere is to make them coincide in three prescribed points (nodes): the origin and the two points symmetrical with respect to the origin, located within the interval [*−r*_0_, *r*_0_].

The position of the nodes can be written as −*r*_0_*t*, 0, and +*r*_0_*t*, where *t* ∈ [0, 1] is a parameter that controls the distance from the origin, at which the wavefront is “matched,” or interpolated. Seeking an explicit expression for *M*, both equations (*W* and *S*) are replaced by their corresponding second-order interpolating polynomials, matching both the wavefront and the fitted sphere at each of the three nodes.

The polynomial interpolating *S* at the three nodes is formed by substituting *r* with *r*_0_*t*.

(7) $$\begin{array}{c} \hat{S}\left(r\right)=W\left(0\right)+\frac{r^{2}}{R+\sqrt{R^{2}-r_{0}^{2}t^{2}}}. \end{array}$$

Both *S*(*r*) and its interpolating polynomial *S*(*r*) take the same values at 0 and ±*r*_0_*t*.

Similarly, the polynomial interpolating *W* at the three nodes is

(8) $$\begin{array}{c} \hat{W}\left(r\right)=W\left(0\right)+\frac{2\Delta \left(t\right)}{r_{0}^{2}}r^{2}, \end{array}$$

where

(9) $$\begin{array}{c} \Delta \left(t\right)≔\sqrt{3}C_{2}^{0}+3\sqrt{5}C_{4}^{0}\left(t^{2}-1\right)+\sqrt{7}C_{6}^{0}\left(10t^{4}-15t^{2}+6\right). \end{array}$$

Well-known formulas for the Lagrange interpolation can be used to calculate $\hat{W}\left(r\right)$; however, it is easier to verify the expression in (9) by taking into account that both *W*(*r*) truncated after the sixth order and its interpolating polynomial *W*(*r*) take the same values at 0 and ±*r*_0_*t*.

As the last step or the derivation, *S* and *W* are equated and solved for *R*.

(10) $$\begin{array}{c} R\left(t\right)=t^{2}\Delta \left(t\right)+\frac{r_{0}^{2}}{4\Delta \left(t\right)}\approx \frac{r_{0}^{2}}{4\Delta \left(t\right)}. \end{array}$$

The term *t*^2^Δ(*t*) can be dropped in case of a human eye, as it is much smaller than the term following it, because *r*_0_ is several millimeters and Δ(*t*) is of an order of microns.

Finally, the formula for the spherical equivalent *M* is as follows:

(11) $$\begin{array}{c} M\left(t\right)=\frac{-1}{R}\left(t\right)=\frac{-4\Delta \left(t\right)}{r_{0}^{2}}=\frac{-4\sqrt{3}C_{2}^{0}-12\sqrt{5}C_{4}^{0}\left(t^{2}-1\right)-4\sqrt{7}C_{6}^{0}\left(10t^{4}-15t^{2}+6\right)}{r_{0}^{2}}. \end{array}$$

**Figure 1:**
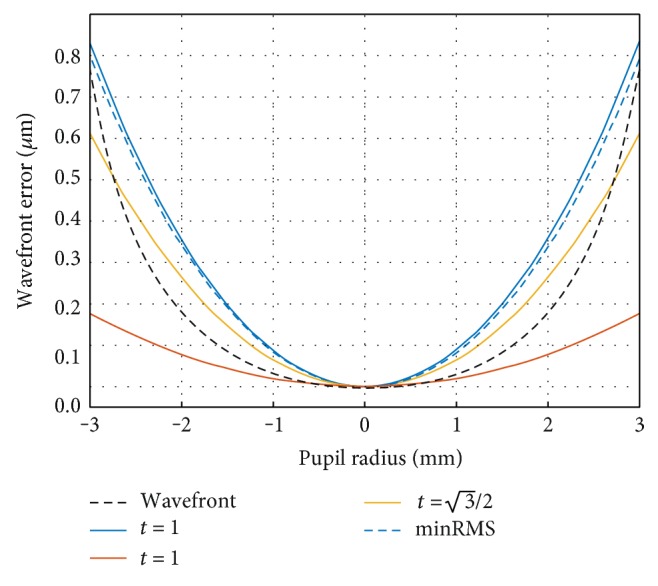
Example profiles of spherical and paraboloid fits (dashed blue line: minRMS; red line: *t* = 0; blue line: *t* = 1; orange line: $t=\sqrt{3}/2$) to radially symmetrical wavefront (represented by the black dotted line, where *C*_2_^0^ = 0.220 *μ*m, *C*_4_^0^ = 0.050 *μ*m, *C*_6_^0^ = 0.0025 *μ*m, and *r*_0_ = 3 mm).

This formula defines a one-parameter family of spherical equivalents: the parameter *t* controls the position of the nodes at which the equivalent sphere matches the wavefront aberration function (Figure 1()), and so, the previously described minRMS and paraxial metrics can be obtained from (11), depending on the value of *t*.

**Figure 2:**
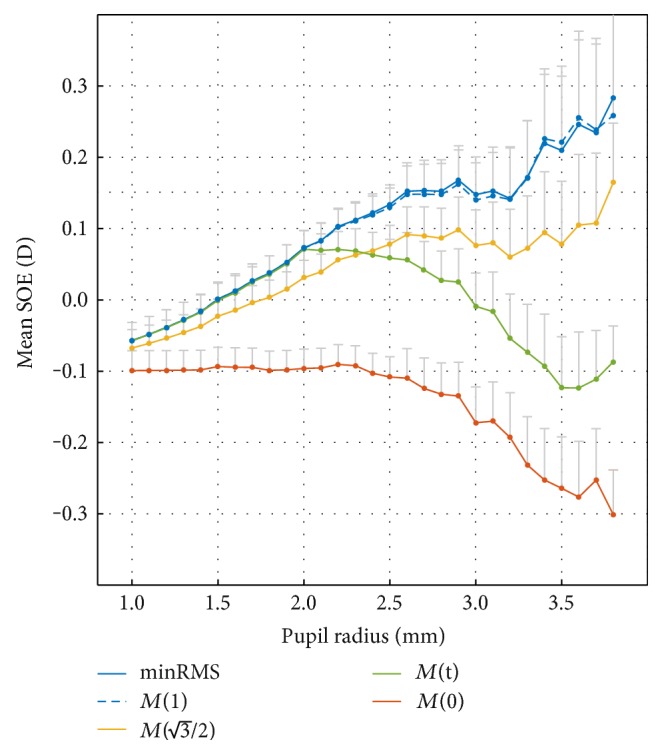
Mean SOE in function of the pupil radius for all 308 subjects of the AHM dataset, and for *t* = 0, 1, $\sqrt{3}/2$, *t*(*r*), and additionally, the minRMS metric. The error bars represent +1 SEM.

(a) When *t* = 0, the sphere is fit at the center of the wavefront, and (11) becomes identical to (2) (paraxial metric).
(b) When *t* = 1, the sphere is fit to the center and extremes of the wavefront (Figure 1()) and (11) corresponds approximately to (1) (minRMS metric).
  (12) $$\begin{array}{c} M\left(1\right)=\frac{-4\sqrt{3}C_{2}^{0}-4\sqrt{7}C_{6}^{0}}{r_{0}^{2}}\approx \frac{-4\sqrt{3}C_{2}^{0}}{r_{0}^{2}}. \end{array}$$
  The approximation by means of dropping the last term is justified by the fact that sixth-order SA is usually very low in human eyes [33]. This approximation is shown in Figure 1() and validated experimentally later (Figure 2()).
(c) When an intermediate value is used, for instance $t=\sqrt{3}/2$, (11) becomes
  (13) $$\begin{array}{c} M\left(\frac{\sqrt{3}}{2}\right)=\frac{-4\sqrt{3}C_{2}^{0}+3\sqrt{5}C_{4}^{0}+36\sqrt{7}C_{6}^{0}}{r_{0}^{2}}. \end{array}$$
  In this case, the nodes $-\sqrt{3}/2$, 0, and $+\sqrt{3}/2$ correspond to the zeros of the cubic Chebyshev polynomial of the second kind, which are known to provide a quasi-optimal set of interpolation nodes [34].

Figure 1 shows the example profiles of spherical and paraboloid fits to a radially symmetrical wavefront, described by (4). All of the profiles have been fixed to coincide at the apex of the wavefront.

The paraxial fit (*t* = 0) matches the wavefront well at the pupil center but does not take into account the shape of the wavefront for intermediate and peripheral areas of the pupil. On the other hand, the minRMS fit (*t* = 1) matches the wavefront well at the edge of the pupil but not at intermediate areas. The Chebyshev fit ($t=\sqrt{3}/2$) matches the wavefront at a predefined, intermediate distance from the center of the pupil.

Given a wavefront described by a set of Zernike coefficients *C*_2_^0^, *C*_4_^0^, and *C*_6_^0^ for a given *r*_0_, (11) can be used to find the optimal value *t*_opt_, such that *M*(*t*_opt_) best approximates the *M*_subjective_. In the present study, this approach is applied to a large database of objective and subjective refractions of real subjects.

The same methodology that was used to analytically derive the equivalent sphere *M* can be extended to take into account the whole spherocylindrical refraction by using power vectors [35]. Because of their orthogonality, the derivation can be performed for two orthogonal directions, corresponding to the higher and lower curvatures of the wavefront. The values of *J*_0_ and *J*_45_ can be obtained in a similar way as *M* in (11), by taking into account the constant coefficients that multiply *ρ*^2^cos(*θ*) and *ρ*^2^sin(*θ*) within each Zernike polynomial.

(14) $$\begin{array}{c} J_{0}\left(t\right)=\frac{-2\sqrt{6}C_{2}^{2}-6\sqrt{10}C_{4}^{2}\left(t^{2}-1\right)-2\sqrt{14}C_{6}^{2}\left(10t^{4}-15t^{2}+6\right)}{r_{0}^{2}}. \end{array}$$

(15) $$\begin{array}{c} J_{45}\left(t\right)=\frac{-2\sqrt{6}C_{2}^{-2}-6\sqrt{10}C_{4}^{-2}\left(t^{2}-1\right)-2\sqrt{14}C_{6}^{-2}\left(10t^{4}-15t^{2}+6\right)}{r_{0}^{2}}. \end{array}$$

The complete spherocylindrical refraction can be obtained using (11), together with (14) and (15), as described by Thibos et al. [35] (see Equation 23 hither).

### 2.4. Numerical Definition of the New Objective Wavefront Refraction Metric

In the previous section, the analytical relationship between a fitted equivalent sphere *M*(*t*_opt_) that optimally approximates *M*_subjective_ was established. In order to find the relationship between the parameter *t*_opt_ and objective refraction pupil radius *r*_0_ (while subjective refraction pupil radius is unknown), the following data processing methodology was applied to the AHM dataset of 308 eyes:

- For each value of pupil radius *r*_0_ from 1.0 mm to 3.8 mm (increment of 0.1 mm) and each value of parameter *t* from 0 to 1 (increment of 0.05), (11) was applied to the Zernike coefficients of every eye to obtain equivalent sphere values.
- The difference between subjective and objective refraction (hereafter called subjective minus objective error (SOE)) was calculated for each eye and the combination of *r*_0_ and *t*.
- For each value of *r*_0_, the mean absolute SOE for all eyes was calculated, and the value of parameter *t* that minimized that mean was selected as *t*_opt_ for that pupil radius.

**Figure 3:**
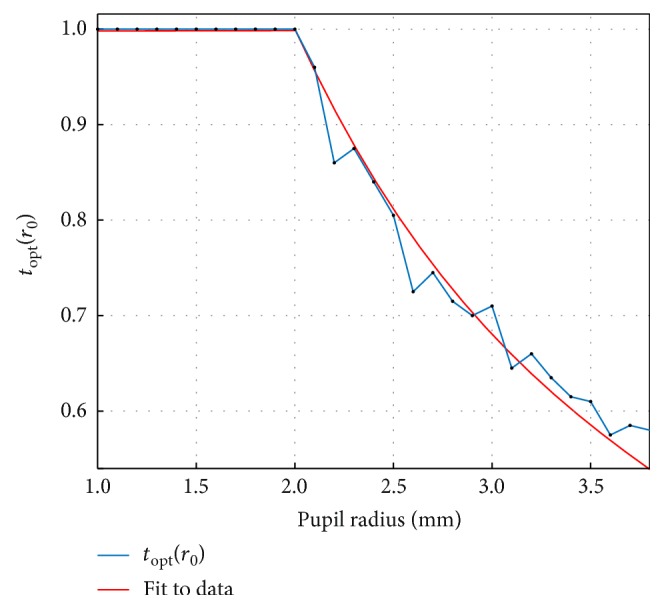
Change of the optimum value of *t* with objective pupil radius *r*_0_ and the segmented fit of the data.

In order to obtain the Zernike coefficients corresponding to each pupil radius in step (a), rescaling [36, 37] was performed from larger to smaller pupil radii [38].  Figure 3 presents the change of *t*_opt_ in function of objective refraction pupil radius *r*_0_ and a segmented fit to the data.

Figure 3() illustrates that for pupils that were small during aberrometry, *t*_opt_ approaches 1 (minRMS metric), while for large pupils, it decreases, and the slope becomes less negative as the pupil gets larger. A segmented fit to the discrete values of the function *t*_opt_(*r*_0_) was performed, and finally, the MTR metric in (17) was obtained.

(16) $$\begin{array}{c} MTR=\frac{-4\sqrt{3}C_{2}^{0}-12\sqrt{5}C_{4}^{0}\left(t^{2}-1\right)-4\sqrt{7}C_{6}^{0}\left(10t^{4}-15t^{2}+6\right)}{r_{0}^{2}}, \end{array}$$

(17) $$\begin{array}{c} t_{opt}\left(r_{0}\right)=\left\{\begin{array}{c} 1,\;r_{0}<2\\ \frac{2}{r_{0}},\;r_{0}\geq 2 \end{array}\right\}, \end{array}$$

which gives the result in diopters when *C*_2_^0^, *C*_4_^0^, and *C*_6_^0^ are expressed in microns and the pupil radius, *r*_0_, is in millimeters.

As described in Section 2.2, the traditional minRMS and paraxial metrics can be obtained from (11), by using fixed values of *t* = 0 and *t* = 1, respectively. The special case where the parameter *t* is a function of *r*_0_, is hereafter referred to as *M*[*t*(*r*)] or, for short, the MTR metric. When the plural form “MTR metrics” is used, the whole family of metrics, where *t* can be either a discrete value or a function of the pupil radius, is being referred to.

## 3. Results

**Figure 4:**
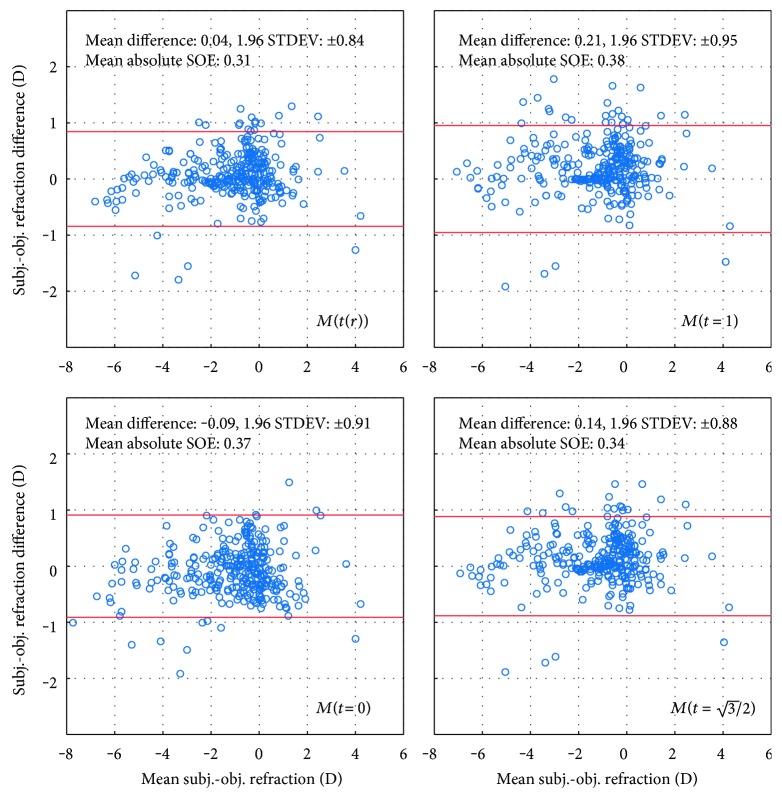
Bland-Altman plots [39] illustrating the accuracy of the prediction of subjective refraction in the AHM dataset for different values of parameter *t*. The data was obtained from WFE without any rescaling of the pupil radii to a common value.

Figure 4 presents the accuracy of the prediction of subjective refraction in the AHM dataset for different values of parameter *t* in form of Bland-Altman plots [39], where the difference between the subjective and objective refraction is plotted in the function of their mean value. Data for the minRMS metric is not shown because the results are practically identical (Figures 1 and 2) to those obtained with the *M*(*t* = 1) metric. No rescaling of Zernike coefficients to a common value of the pupil radius was performed. Each data point represents the SOE value of a corresponding eye, calculated from the Zernike coefficients of the natural pupil radius.

Figure 2 shows the mean SOE as a function of the pupil radius for all 308 subjects of the AHM dataset, for *t* = 0, 1, and $\sqrt{3}/2$, *t*_opt_ = *f*(*r*_0_), and additionally, the classic minRMS metric.

**Figure 5:**
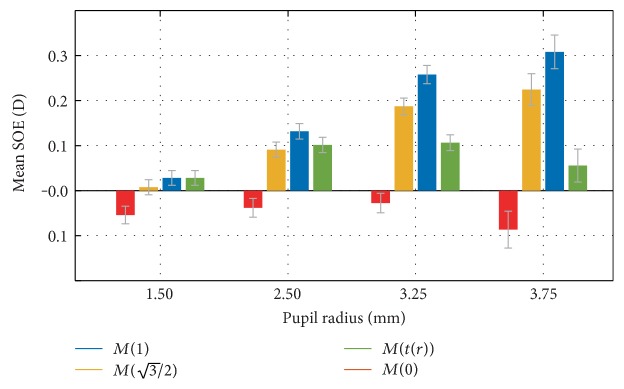
Application of the four MTR metrics to an independent IND dataset (Indiana in Table 1). The error bars represent ±1 SEM.

Finally, in order to validate the results using an independent dataset, Figure 5 shows the mean SOE values calculated using the four MTRs for *t* = 0, $\sqrt{3}/2$, and 1 and *t*_opt_ = *f*(*r*_0_) in the IND dataset of 100 left and 100 right eyes (Table 1). This dataset is called independent because it has not been included in the numerical definition of the MTR metric described in Section 2.1.

As described in Section 2.1, the eyes in the IND dataset were paralyzed using 0.5% cyclopentolate, and optimally corrected for astigmatism. Their hyperfocal points were conjugated with the retina with trial lenses. Consequentially the values of *M*_subjective_ were 0D for this dataset, and the mean SOE in Figure 5 represents the objective spherical equivalent values with the opposite sign. Even though the 100 left and 100 right eyes in the IND dataset cannot be treated as 200 independent eyes, there were no significant differences between the results obtained with the 4 metrics when they were applied to the subsets of 100 left and 100 right eyes separately.

## 4. Discussion

The present study presents an analytical derivation of a new, general formula (11) for the calculation of the spherical equivalent from the WFE. The formula includes the paraxial metric and a close approximation of the minRMS metric (12) as particular cases, where the value of the parameter *t* is a scalar number (0 and 1, resp.). Additionally, a relation between the optimum value of the parameter *t* and the objective refraction pupil radius *r*_0_ is presented as the function *t*_opt_(*r*_0_) ((17), illustrated in Figure 3). This function was obtained empirically from subjective refraction data from 308 eyes, measured following standard clinical protocols and under standard illumination conditions.

The Bland-Altman plots in Figure 4 show that for natural pupil radii (without rescaling the WFE), the mean difference between the values of subjective and objective refraction was the smallest for the MTR metric, although not by a large margin.

Figure 2 presents the mean intersubject SOE in the function of the pupil radius, which was obtained by rescaling [36, 37] the WFE before calculating the metrics. For small pupil radii (up to 2 mm), the HOA are small and do not noticeably affect the objective refraction, so that all of the metrics give similar results. For example, for *r*_0_ = 1 mm, the difference between predictions of subjective refraction between the minRMS and paraxial metrics is merely 0.05D. For pupil radii up to 2 mm, the minRMS metric predicts subjective refraction slightly better than the paraxial metric. For such small pupils, the absolute SOE is similar for both the minRMS and paraxial metrics, and both predict subjective refraction better than the clinical precision of 0.25D.

Furthermore, Figure 2 indicates that rescaling the wavefront to correspond to the radius of 1.5 mm and applying the minRMS metric can be very successfully used to calculate refraction. This result is in agreement with results obtained by others [6, 26, 40] who found that Zernike refraction is a good predictor of subjective refraction when SA is low. Indeed, (17) indicates that *t*_opt_ · *r*_0_ = 2 for *r*_0_ ≥ 2 mm and the MTR metric corresponds to a sphere fitted to the wavefront at 0 and ±2 mm. This means that it predicts a similar equivalent sphere as the one obtained by the minRMS metric to a wavefront rescaled to correspond to a pupil radius of 2 mm [6]. In this case, the SOE calculated for the IND dataset was 0.06 ± 0.03D, which is practically the same as the mean SOE for the MTR metric without rescaling. In Figure 5, it can be seen that its mean SOE indeed does not exceed 0.1D, even for a large pupil radii of 3.75 mm.

It is important to note, however, that the SOE of the paraxial metric *M*(*t =* 0) remains constant at −0.1D for pupil radii from 1 mm to 2.5 mm. This possibly reveals an empirical calibration offset favoring the minRMS metric in aberrometer devices and demonstrates that the calibration of the apparatus used to obtain the WFE data can play a very important role in the determination of the function *t*_opt_(*r*_0_), shown in Figure 3.

For large pupil radii, the refraction calculated using the paraxial metric *M*(*t =* 0) is not expected to change with pupil radius (as it is based on paraxial curvature matching to the pupil center), and its increase in the hyperopic direction for *r*_0_ *≥* 2 mm indicates that subjective spherical equivalent slightly changes with the pupil radius. We found this change to be very small (~0.1D), and it should not affect vision in any way. Charman et al. [41] found a similar effect of the decrease of refraction with pupil size, but it was so small that they concluded that refraction practically did not change. This may explain why paraxial refraction usually gives results in good agreement with subjective refraction for large pupil radii [7, 15]. Within the same range of *r*_0_, the *M*(*t* = 1) metric suffers from a myopic increase of SOE. This trend can be predicted from (12), by taking into account that *C*_4_^0^ and *C*_6_^0^ spherical aberration coefficients increase their values exponentially with pupil radius and their average values are positive in the AHM database, which is usually the case for normal eyes [30]. For pupil radii over 2 mm, there are clear differences in the predictions of subjective refraction depending on the value of *t*. In particular, for *r* > 3.5 mm, the difference can be more than 0.5D, which is clinically significant.

## 5. Conclusions

The present study indicates that for pupil radii less than 2 mm, all of the wavefront refraction metrics are similarly accurate in predicting the equivalent sphere (mean SOE < 0.1D). For large pupil radii, however, the mean absolute SOE can increase beyond 0.25D for traditional metrics, which is clinically significant. This is caused by two factors. First, the effects of SA for large pupil radii cause the minRMS objective refraction to become significantly too myopic. Secondly, subjective refraction increases slightly in the myopic direction for large pupil radii, which increases the SOE for paraxial objective refraction in the hyperopic direction (Figure 2()).

The new MTR objective wavefront refraction metric (16-17) is designed to depend on the objective refraction pupil radius, applying more or less weight to the SA coefficients in the function of *r*_0_.

The benefit of this methodology lies in the fact that the MTR metric allows clinicians to obtain an accurate clinical spherical equivalent value without rescaling/refitting of the wavefront coefficients. It has the potential to be developed into a metric which will be able to predict full spherocylindrical refraction for the desired illumination conditions and corresponding pupil size. Several formulas can be applied to determine the pupil size from illumination, subject's age, and type of task to be performed [42].

## Figures and Tables

**Table 1:** Summary of datasets of eyes included in the study.

Dataset	Number of eyes OD/OS (total)	Mean age	Cycloplegia	Pupil diameter	Aberrometer
Army [25] (A)	47/47(94)	29.9 ± 7.6	No	5.0 mm	COAS
Houston [26] (H)	20/20(40)	26.4 ± 7.7	Tropicamide 1%	7.4 ± 0.5 mm	COAS
Murcia [20] (M)	87/87(174)	35.0 ± 12.4	No	5.5 ± 0.9 mm	irx3
(AHM)	154 subjects, 308 eyes				
Indiana [7] (IND)	100/100(200)	26.1 ± 5.6	Cyclopentolate 0.5%	>7.5 mm (140 eyes) >6.0 mm (60 eyes)	Custom
